# Serum and Synovial Fluid Interleukin-6 for the Diagnosis of Periprosthetic Joint Infection

**DOI:** 10.1038/s41598-017-01713-4

**Published:** 2017-05-04

**Authors:** Kai Xie, Kerong Dai, Xinhua Qu, Mengning Yan

**Affiliations:** Shanghai Key Laboratory of Orthopaedic Implants, Department of Orthopaedic Surgery, Shanghai Ninth People’s Hospital, Shanghai Jiao Tong University School of Medicine, Shanghai, PR China

## Abstract

A gold standard for diagnosis of periprosthetic joint infection (PJI) has not yet been established. The objective of this study was to evaluate the diagnostic value of serum and synovial fluid interleukin (IL)-6 levels for PJI. The MEDLINE and EMBASE databases were searched for studies describing PJI diagnosis using serum and synovial fluid IL-6 and published between January 1990 and October 2016. Seventeen studies were included in the analysis. The pooled sensitivities of serum and synovial fluid IL-6 were 0.72 (95% confidence interval [CI]: 0.63–0.80) and 0.91 (95% CI: 0.82–0.96), respectively. The pooled specificities of serum and synovial fluid IL-6 were 0.89 (95% CI: 0.77–0.95) and 0.90 (95% CI: 0.84–0.95), respectively. The pooled diagnostic odds ratios (DORs) of serum and synovial fluid IL-6 were 20 (95% CI: 7–58) and 101 (95% CI: 28–358), respectively, and the pooled areas under the curve (AUCs) were 0.83 (95% CI: 0.79–0.86) and 0.96 (95% CI: 0.94–0.98), respectively. Synovial fluid IL-6 had high diagnostic value for PJI. Although serum IL-6 test was less sensitive than synovial fluid IL-6 test, it may be regularly prescribed for patients with prosthetic failure owing to its high specificity.

## Introduction

Periprosthetic joint infection (PJI) is a severe complication of arthroplasty and causes unrelieved pain and joint dysfunction. Although standardised perioperative management has lowered the risk of hip and knee PJI to less than 2%, PJI is still a significant cause of revision surgery^1–5^. In contrast to aseptic loosening, PJI is often associated with a two-stage revision procedure and long-term antibiotic therapy. Given these adverse implications, an accurate diagnosis method for PJI is valuable in arthroplasty surgery. In the past decades, various diagnostic methods and guidelines have been established for diagnosing PJI. Unfortunately, in the absence of a gold standard test, it is still challenging to differentiate PJI from aseptic loosening^6^. A previous prospective cohort study reported that 25% of PJI patients were misdiagnosed with aseptic loosening in the first year after arthroplasty^7^. Periprosthetic tissue culture and histological examination are reliable diagnostic methods for PJI detection^8^; however, neither method can guide the operative decision before revision surgery.

Inflammatory biomarkers in the serum and synovial fluid can be evaluated before surgery^8^. Traditional biomarkers such as serum white cell count (WCC) and C-reactive protein (CRP) have limited diagnostic accuracy for PJI detection^9, 10^. Previous meta-analysis indicated that the sensitivity of serum WCC for PJI detection is 0.45^9^. The pooled sensitivity and specificity of serum CRP are 0.82 and 0.77, respectively^10^. Recently, the diagnostic value of interleukin-6 (IL-6) for PJI detection was investigated. IL-6 is produced by lymphoid and non-lymphoid cells, and it participates in the inflammatory response^11^. Serum IL-6 levels increase with trauma, infection, and surgery^11–13^. In patients with aseptic prosthetic loosening, IL-6 levels decrease to the normal level within 48 h after arthroplasty^13^. However, following infection, IL-6 activates the release of CRP^14^. Therefore, the increase of IL-6 precedes that of CRP after infection; thus, IL-6 may be a more sensitive marker for PJI.

The previous meta-analysis summarised the results of three studies and showed that high serum IL-6 level strongly indicates PJI^9^. Following this study, the diagnostic capacity of serum and synovial fluid IL-6 for PJI has been widely evaluated; however, there were some discrepancies in the results^15–31^. The objective of the present meta-analysis was to estimate the value of serum and synovial fluid IL-6 assessments for PJI diagnosis.

## Results

### Search Results

**Figure 1:**
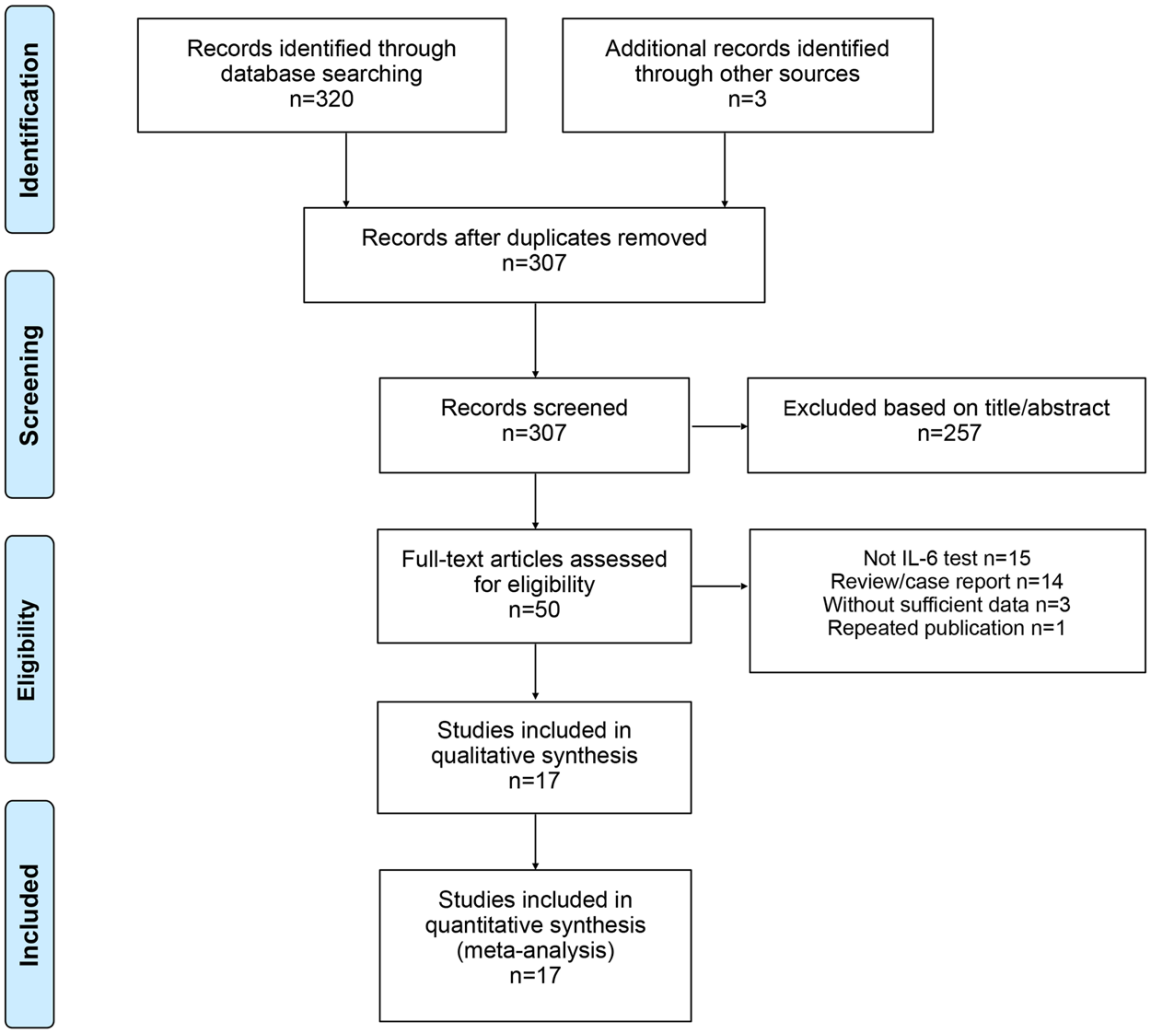
Flow diagram for study selection.

A total 323 articles were identified following the database and bibliography search. After further screening, 257 articles were excluded, and 33 articles were excluded after full-text evaluation (Fig. 1). Seventeen articles were included in the current analysis (Table 1). Nine studies were conducted in the United States, three in Germany, and one study each in Sweden, Argentina, Austria, United Kingdom, and Egypt. Nine articles described the diagnostic accuracy of serum IL-6 for PJI, and six articles focused on synovial fluid IL-6 test. Two additional studies investigated both serum and synovial fluid IL-6 assessments for PJI diagnosis. All studies included in the current meta-analysis were prospective studies. Consecutive patient enrolment was used in four studies, while the others did not indicate the type of patient enrolment. Three studies included patients with shoulder arthroplasty, while the others focused on knee and/or hip arthroplasty. The number of participants ranged from 35 to 120, and the mean age ranged from 58 to 72 years. The cut-off values of serum and synovial fluid IL-6 levels for PJI detection in the selected studies ranged from 2.6 to 10.4 pg/mL and 359.3 to 13,350 pg/mL, respectively. All studies were evaluated using the QUADAS tool, and showed moderate to high quality.

**Table 1:** Characteristics of the studies in meta-analysis for the diagnosis of PJI using serum and synovial fluid IL-6.

Study	Country	Patients Number	Mean Age (y)	Study design, Enrollment	Excluded inflammatory disease	Cut-off	Sample Part	Ref. Standard	QUADAS
**Serum IL-6**									
Di Cesare *et al*.^15^	USA	58	63	Prospective NA	Y	10.0 pg/ml	Hip; Knee	H; M	14
Bottner *et al*.^16^	USA	78	64	Prospective NA	N	12.0 pg/ml	Hip; Knee	H; M	14
Buttaro *et al*.^18^	Argentina	69	68	Prospective NA	Y	10.0 pg/ml	Hip	H; M	14
Worthington *et al*.^20^	UK	46	72	Prospective NA	Y	9.0 pg/ml	Hip	M	14
Abou EI-Khier *et al*.^22^	Egypt	40	58	Prospective NA	Y	10.4 pg/ml	Hip; Knee	IOF; H; M	13
Glehr *et al*.^23^	Austria	84	NA	Prospective NA	Y	4.7 pg/ml	Hip; Knee	H; M	14
Gollwitzer *et al*.^24^	Germany	35	70	Prospective consecutive	Y	1.89 pg/ml	Hip; Knee	IOF; H; M	13
Grosso *et al*.^26^	USA	69	62	Prospective consecutive	Y	5.0 pg/ml	Shoulder	IOF; M	13
Randau *et al*.^27^	Germany	120	68	Retrospective NA	NA	2.6 pg/ml	Hip; Knee;	IOF; H; M	13
Villacis *et al*.^28^	USA	34	64	Prospective NA	Y	10.0 pg/ml	Shoulder	H; M	14
Ettinger *et al*.^29^	Germany	98	67	Prospective NA	Y	5.12 ng/ml	Hip; Knee; Shoulder	IOF; H; M	13
**Synovial fluid IL-6**									
Nilsdotter-Augustinsson *et al*.^17^	Sweden	85	NA	Prospective Consecutive	N	10000 pg/ml	Hip	IOF; M	13
Deirmengain *et al*.^19^	USA	51	65	Prospective NA	N	13350 pg/ml	Hip; Knee	IOF; M	13
Jacovides *et al*.^21^	USA	73	65	Prospective NA	NA	4270 pg/ml	Hip; Knee	IOF; M	13
Gollwitzer *et al*.^24^	Germany	35	70	Prospective Consecutive	Y	1896.56 pg/ml	Hip; Knee	IOF; H; M	13
Deirmengain *et al*.^25^	USA	95	67	Prospective NA	N	2300 pg/ml	Hip; Knee	IOF; H; M	13
Randau *et al*.^27^	Germany	120	68	Prospective NA	NA	2100 pg/ml	Hip; Knee	IOF; H; M	13
Frangiamore *et al*.^30^	USA	32	61	Prospective Consecutive	NA	359.3 pg/ml	Shoulder	IOF; H; M	13
Frangiamore *et al*.^31^	USA	90	64	Prospective NA	N	8671 pg/ml	Hip; Knee	IOF; H; M	13

H: Histological examination; IOF: Intraoperative finding; M: Microbiological or Laboratory examination; NA: Not available; Ref. Standard: Reference standard.

### Diagnostic Accuracy

**Figure 2:**
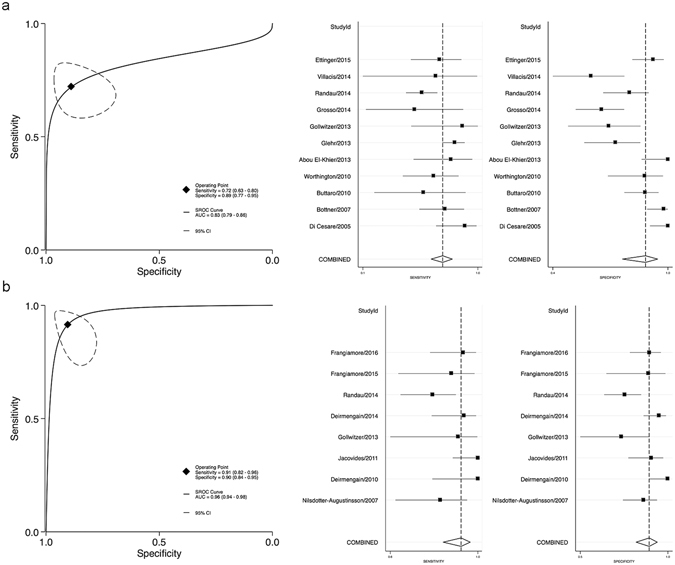
Summary receiver-operating characteristic curves and forest plots for serum (**a**) and synovial fluid interleukin (IL)-6 (**b**).

Pooled sensitivity, specificity, area under the curve (AUC), and diagnostic odds ratio (DOR) results are shown in Fig. 2. The pooled sensitivity and specificity for PJI diagnosis using serum IL-6 were 0.72 (95% CI: 0.63–0.80) and 0.89 (95% CI: 0.77–0.95), respectively. The pooled sensitivity and specificity for PJI diagnosis using synovial fluid IL-6 were 0.91 (95% CI: 0.82–0.96) and 0.90 (95% CI: 0.84–0.95), respectively. The pooled DORs for PJI diagnosis using serum and synovial fluid IL-6 were 20 (95% CI: 7–58) and 101 (95% CI: 28–358), respectively. The pooled AUCs for serum and synovial fluid IL-6 tests were 0.83 (95% CI: 0.79–0.86) and 0.96 (95% CI: 0.94–0.98), respectively. Heterogeneity was evaluated by I^2^. The I^2^ of serum IL-6 was 81, indicating substantial variation among the included studies. No heterogeneity was found for synovial fluid IL-6 test (I^2^ = 0).

### Evaluation of the Clinical Utility

**Figure 3:**
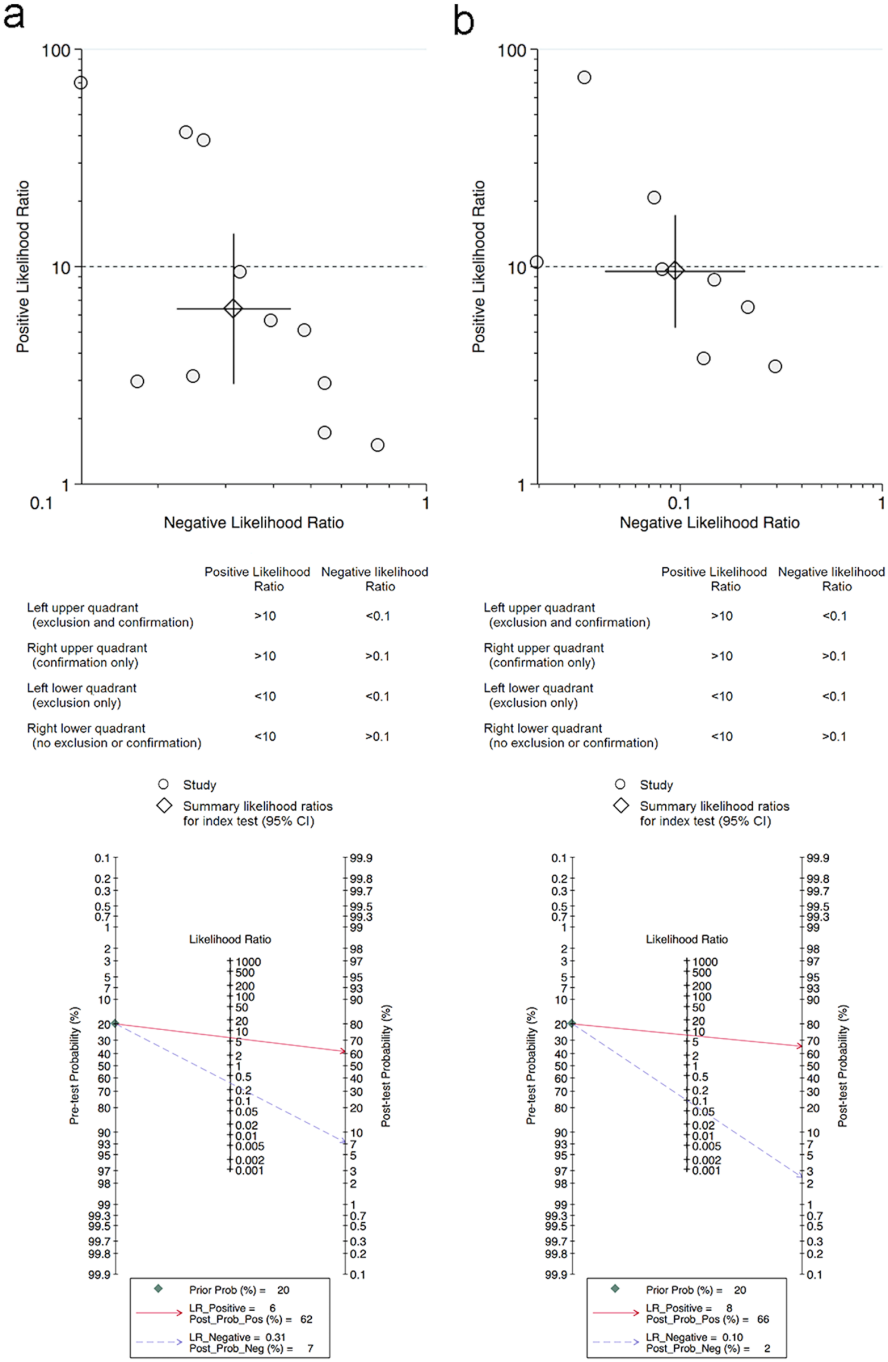
Likelihood ratio scatter diagrams and post-test probabilities for serum (**a**) and synovial fluid IL-6 (**b**).

The positive likelihood ratio (PLR) and negative likelihood ratio (NLR) of serum IL-6 for PJI diagnosis were 6.4 (95% CI: 2.9–14.1) and 0.31 (95% CI: 0.22–0.44), respectively, while those of synovial fluid IL-6 were 9.5 (95% CI: 5.3–17.2) and 0.09 (95% CI: 0.04–0.21), respectively (Fig. 3). Likelihood ratios and pre-test probabilities were used to calculate post-test probabilities. Based on the low prevalence of PJI, 20% pre-test probabilities were used in current study. The post-test probability of PJI was 7% and 2% for serum and synovial IL-6 tests, respectively, indicating negative results (Fig. 3).

### Subgroup Analysis

Results of the subgroup analysis are presented in Table 2. Both serum and synovial fluid IL-6 tests showed reliable diagnostic accuracy for PJI after hip and knee arthroplasty. The sensitivities of serum and synovial fluid IL-6 for hip and/or knee PJI diagnosis were 0.74 (95% CI: 0.63–0.80) and 0.92 (95% CI: 0.82–0.97), respectively. The specificities of serum and synovial fluid IL-6 for hip and/or knee PJI diagnosis were 0.92 (95% CI: 0.79–0.97) and 0.91 (95% CI: 0.83–0.95), respectively. The current analysis also showed that the cut-off value could influence the diagnostic accuracy of both serum and synovial fluid IL-6 tests. When the cut-off value was greater than 10 and 2,300 pg/mL for serum and synovial fluid IL-6 tests, respectively, the diagnostic accuracy was improved. The diagnostic accuracy of synovial fluid IL-6 was not affected by the presence of inflammatory diseases. In studies that included patients with inflammatory diseases, the pooled sensitivity and specificity of synovial fluid IL-6 were 0.92 and 0.93, respectively.

**Table 2:** Subgroup analysis of serum and synovial fluid IL-6 for PJI diagnosis.

	Number of Studies	Sensitivity (95% CI)	Specificity (95% CI)	AUC (95% CI)	PLN (95% CI)	NLR (95% CI)	DOR (95% CI)
**Serum IL-6**							
Overall studies	11	0.72 (0.63–0.80)	0.89 (0.77–0.95)	0.83 (0.79–0.86)	6.4 (2.9–14.1)	0.31 (0.22–0.44)	20 (7–58)
Hip and/or knee	8	0.74 (0.64–0.82)	0.92 (0.79–0.97)	0.86 (0.83–0.89)	9.4 (3.3–26.8)	0.28 (0.19–0.41)	34 (9–124)
Excluded inflammatory disease	8	0.77 (0.68–0.84)	0.88 (0.70–0.95)	0.82 (0.78 + 0.85)	6.2 (2.4–16.6)	0.26 (0.17–0.39)	24 (7–84)
**Number of patient**							
$\ge$60	6	0.69 (0.57–0.78)	0.86 (0.74–0.93)	0.81 (0.78–0.85)	5.0 (2.5–9.9)	0.36 (0.25–0.52)	14 (5–35)
<60	5	0.77 (0.63–0.87)	0.90 (0.67–0.98)	0.83 (0.79–0.86)	7.7 (1.9–31.0)	0.26 (0.15–0.45)	30 (5–181)
**Cut-off**							
$\ge$10 pg/ml	5	0.77 (0.64–0.87)	0.98 (0.87–1.00)	0.91 (0.88–0.93)	47.7 (5.2–441.9)	0.23 (0.14–0.38)	208 (17–2548)
<10 pg/ml	6	0.70 (0.59–0.79)	0.80 (0.70–0.87)	0.81 (0.78–0.84)	3.4 (2.3–5.2)	0.38 (0.27–0.53)	9 (5–17)
**Synovial fluid IL-6**							
Overall studies	8	0.91 (0.82–0.96)	0.90 (0.84–0.95)	0.96 (0.94–0.98)	9.5 (5.3–17.2)	0.09 (0.04–0.21)	101 (28–358)
Hip and/or knee	7	0.92 (0.82–0.97)	0.91 (0.83–0.95)	0.97 (0.95–0.98)	9.8 (5.0–19.2)	0.09 (0.03–0.22)	115 (26–509)
**Excluded inflammatory disease**							
Yes and NA	4	0.91 (0.72–0.97)	0.84 (0.75–0.91)	0.92 (0.89–0.94)	5.8 (3.3–10.1)	0.11 (0.03–0.38)	52 (10–280)
No	4	0.92 (0.81–0.97)	0.93 (0.87–0.96)	0.97 (0.95–0.98)	13.0 (6.7–25.1)	0.08 (0.03–0.22)	153 (37–629)
**Number of patient**							
$\ge$80	4	0.87 (0.76–0.93)	0.89 (0.80–0.94)	0.94 (0.92–0.96)	7.8 (4.0–14.9)	0.15 (0.07–0.30)	53 (15–184)
<80	4	0.97 (0.76–1.00)	0.92 (0.77–0.97)	0.98 (0.97–0.99)	11.8 (3.8–36.9)	0.03 (0.00–0.33)	358 (17–7589)
**Cut-off**							
$\ge$2300 pg/ml	4	0.96 (0.80–0.99)	0.93 (0.88–0.96)	0.97 (0.95–0.98)	14.2 (7.3–27.8)	0.05 (0.01–0.24)	302 (39–2342)
<2300 pg/ml	4	0.86 (0.74–0.93)	0.84 (0.76–0.90)	0.92 (0.89–0.94)	5.4 (3.3–8.9)	0.17 (0.08–0.33)	32 (11–94)

### Assessment of Publication Bias

**Figure 4:**
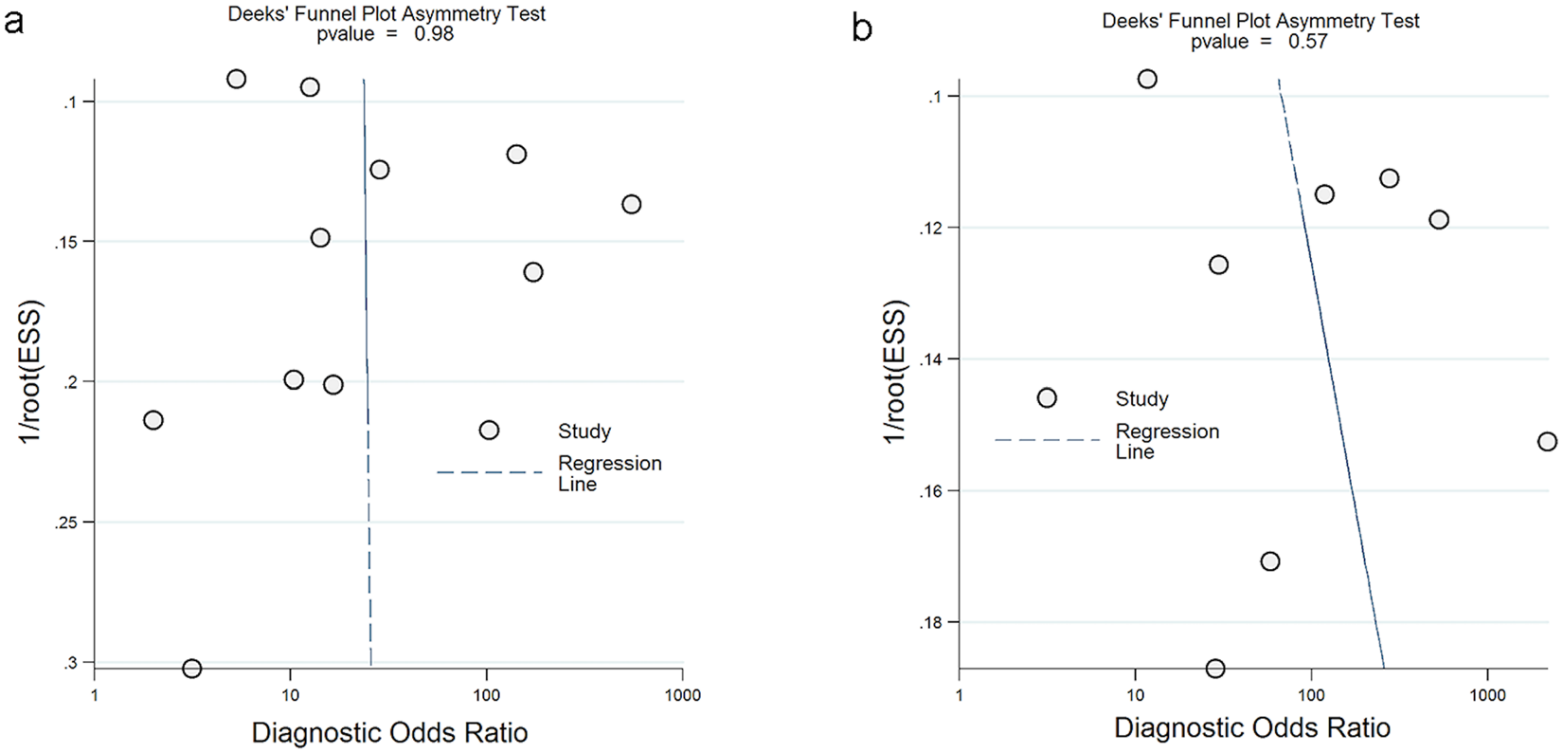
Funnel plots for the included studies: serum IL-6 (**a**) and synovial fluid IL-6 (**b**).

No potential publication bias was identified for studies investigating serum (*p* = 0.98) and synovial fluid IL-6 (*p* = 0.57) tests. The results are presented in Fig. 4.

## Discussion

The current meta-analysis indicated that synovial fluid IL-6 can be used for the diagnosis of PJI after joint arthroplasty. Although serum IL-6 is less sensitive than synovial fluid IL-6, it is one of the best serum biomarkers for PJI detection. Because of its reliable post-test probability, serum IL-6 assessment should be included as a regular test for patients with prosthetic failure.

**Figure 5:**
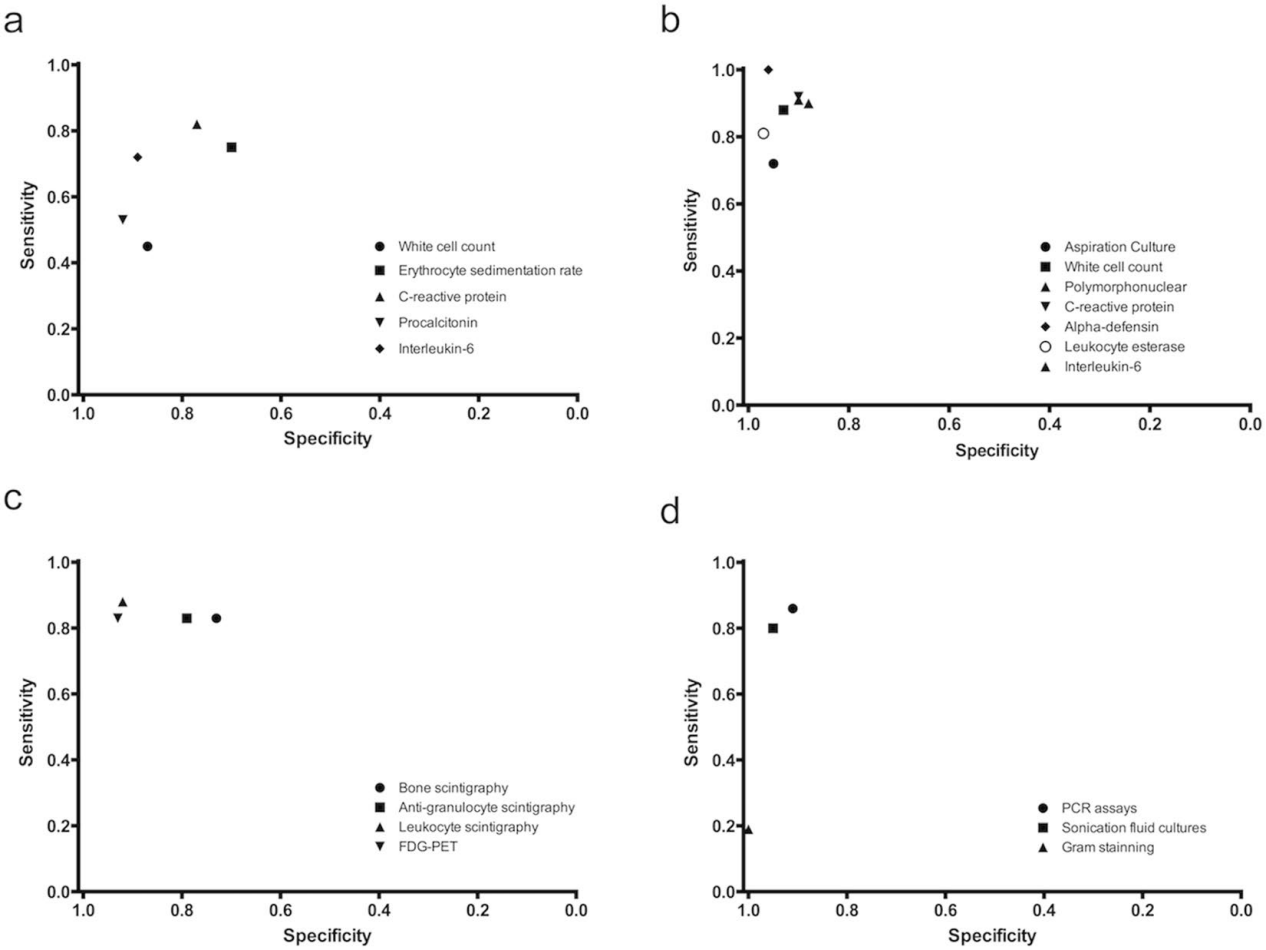
Scatter diagrams for the diagnostic accuracy of the IL-6 test, as assessed by the meta-analysis: serum biomarkers (**a**); synovial fluid biomarkers and aspiration culture (**b**); nuclear medicine (**c**); and tests with biopsy (**d**).

Treatment for PJI involves intensive debridement, one- or two-stage implantation, and long-term antimicrobial therapy; in contrast, a generic revision procedure is typically sufficient to treat aseptic loosening^6^. Thus, a precise diagnosis is necessary for surgeons to lay out the operation plan. However, PJI is difficult to diagnose before revision surgery in the absence of uniform criteria and a gold standard test. In the past decade, several meta-analyses have been performed to assess the diagnostic accuracy of various established tests for PJI detection (Table 3 and Fig. 5)^9, 10, 32–43^. Traditionally, periprosthetic tissue culture, which has a positive detection rate of 0.70–0.90, is regarded as the gold standard test for PJI diagnosis^8, 44–46^. Two prior meta-analyses have demonstrated that sonication and polymerase chain reaction can increase the diagnostic accuracy of prosthetic biopsy^41, 42^. Unfortunately, these intraoperative tests cannot be performed before revision surgery.

**Table 3:** Diagnostic value of different diagnostic method for the diagnosis of PJI.

Diagnostic method	Study	Sensitivity 95% CI	Specificity 95% CI	PLR 95% CI	NLR 95% CI	DOR 95% CI	AUC 95% CI
**Serum biomarkers**							
White cell count	Berbari *et al*.^9^	0.45 (0.41–0.49)	0.87 (0.85–0.89)	NA	NA	4.40 (2.90–6.60)	NA
ESR	Berbari *et al*.^9^	0.75 (0.72–0.77)	0.70 (0.68–0.72)	NA	NA	7.20 (4.70–10.90)	NA
C-reactive protein	Yuan *et al*.^10^	0.82) (0.80–0.84)	0.77 (0.76–0.78)	3.66 (2.92–4.59)	0.26 (0.20–0.33)	17.01 (11.38–25.44)	0.88 (0.86–0.89)
Procalcitonin	Xie *et al*.^32^	0.53 (0.24–0.80)	0.92 (0.45–0.99)	6.80 (1.00–48.10)	0.51 (0.31–0.84)	13.00 (3.00–70.00)	0.76 (0.72–0.80)
Interleukin-6	Current study	0.72 (0.63–0.80)	0.89 (0.77–0.95)	6.40 (2.90–14.10)	0.31 (0.22–0.44)	20.00 (7.00–58.00)	0.83 (0.79–0.86)
**Synovial fluid biomarkers and Aspiration culture**							
Aspiration culture	Qu *et al*.^33^	0.72 (0.65–0.78)	0.95 (0.93–0.97)	15.3 (10.6–22.1)	0.29 (0.23–0.38)	52.00 (31.00–86.00)	0.94 (0.92–0.96)
White cell count	Qu *et al*.^34^	0.88 (0.81–0.93)	0.93 (0.88–0.96)	13.30 (7.70–22.80)	0.13 (0.08–0.21)	103.00 (54.00–197.00)	0.96 (0.94–0.98)
Polymorphonuclear	Qu *et al*.^34^	0.90 (0.84–0.93)	0.88 (0.83–0.92)	7.60 (4.90–11.70)	0.12 (0.07–0.19)	64.00 (27.00–149.00)	0.95 (0.93–0.96)
C-reactive protein	Wang *et al*.^35^	0.92 (0.86–0.96)	0.90 (0.87–0.93)	9.00 (6.15–13.16)	0.10 (0.06–0.18)	101.40 (48.07–213.93)	0.97 NA
Alpha-defensin	Wyatt *et al*.^36^	1.00 (0.82–1.00)	0.96 (0.89–0.99)	27.0 (9.0–80.6)	0.00 (0.00–0.22)	NA	0.99 (0.98–1.00)
Leukocyte esterase	Wyatt *et al*.^36^	0.81 (0.49–0.95)	0.97 (0.82–0.99)	23.90 (3.80–152.10)	0.19 (0.06–0.66)	NA	0.97 (0.95–0.98)
Interleukin-6	Current study	0.91 (0.82–0.96)	0.90 (0.84–0.95)	9.50 (5.40–17.20)	0.09 (0.04–0.21)	101.00 (28.00–358.00)	0.96 (0.94–0.98)
**Nuclear medicine**							
Bone scintigraphy	Ouyang *et al*.^37^	0.83 (0.72–0.90)	0.73 (0.65–0.80)	3.10 (2.40–4.10)	0.23 (0.14–0.38)	14.00 (7.00–26.00)	0.85 (0.81–0.87)
Anti-granulocyte Scintigraphy	Xing *et al*.^38^	0.83 (0.79–0.87)	0.79 (0.75–0.83)	3.56 (2.42–5.23)	0.26 (0.19–0.37)	18.76 (10.45–33.68)	0.88 NA
Leukocyte scintigraphy	Verberne *et al*.^39^	0.88 NA	0.92 NA	NA	NA	NA	NA
FDG-PET	Verberne *et al*.^39^	0.86 NA	0.93 NA	NA	NA	NA	NA
**Other tests with biopsy**							
Frozen section histopathology	Tsaras *et al*.^40^	NA	NA	12.00 (8.40–17.20)	0.23 (0.15–0.35)	54.7 (31.2–95.7)	NA
PCR assays	Qu *et al*.^34^	0.86 (0.77–0.92)	0.91 (0.81–0.96)	9.10 (4.60–18.20)	0.16 (0.10–0.25)	59.00 (29.00–118.00)	0.94 (0.91–0.95)
Sonication fluid cultures	Zhai *et al*.^42^	0.80 (0.74–0.84)	0.95 (0.90–0.98)	17.20 (7.60–38.70)	0.21 (0.17–0.27)	81.00 (35.00–186.00)	0.89 (0.86–0.91)
Gram staining	Ouyang *et al*.^43^	0.19 (0.12–0.27)	1.00 (0.99–1.00)	41.60 (15.50–111.20)	0.82 (0.75–0.89)	51.00 (18.00–140.00)	0.89 (0.86–0.91)

If one diagnostic method was reported by more than one meta-analyses, the most detailed and/or recent one was included in Table 3. ESR: Erythrocyte sedimentation rate; FDG-PET: 18F-fluoro-2-deoxyglucose positron emission tomography. PCR: Polymerase chain reaction.

Radiography is the most basic test prescribed for patients with joint pain after arthroplasty. Implant migration with transcortical sinus tracts is a reliable indicator for PJI^6^. However, septic hip prosthetic loosening typically shows normal radiographic findings^47^. Because metal-related artefacts are used in these patients, computed tomography and magnetic resonance imaging cannot be used for PJI diagnosis. Nuclear medicine appears to have a high sensitivity for PJI detection. The pooled sensitivity and specificity of leukocyte scintigraphy were 0.88 and 0.92, respectively, and those for fluorodeoxyglucose positron emission tomography were 0.86 and 0.93, respectively. The disadvantages of nuclear medicine for PJI diagnosis are high cost and radiation dose^8^.

Serum biomarkers are commonly utilised to detect infections worldwide. According to the guidelines specified by the Infectious Diseases Society of America (IDSA), the erythrocyte sedimentation rate and CRP should be evaluated for all patients suspected as having PJI^8^. However, serum biomarker levels can be easily influenced by antibiotic therapy, systemic infection, trauma, and surgery^48^; thus, these biomarkers may indicate inflammation rather than infection. According to a previous study, procalcitonin (PCT) is the most specific serum biomarker of PJI with a specificity of 0.92^32^.

Recently, several studies have investigated the diagnostic capacity of serum and synovial fluid IL-6 for PJI. IL-6 is a 212-amino acid interleukin encoded by a single gene mapped to chromosome 7 in humans^49^. The correlation between high levels of IL-6 in body fluids and local acute bacterial infection was reported in 1989^50^. The level of serum IL-6 is typically less than 10 pg/mL in healthy adults^51^. IL-6 is an anti-inflammatory cytokine and a mediator of infection response^52^. It is considered the major mediator of acute phase protein production by hepatocytes^53^. Serum CRP level increases after 4–6 h of IL-6 stimulation^54^, indicating that IL-6 is a relatively sensitive biomarker of early-stage immune response. As reported in a previous meta-analysis that assessed the findings of three studies, the pooled sensitivity and specificity of serum IL-6 were 0.97 and 0.91, respectively for PJI detection^9^. This remarkable diagnostic accuracy is inconsistent with the evidence presented in the current report. In the present meta-analysis, 11 studies that assessed serum IL-6 levels from 2005 to 2015 were included. The present study demonstrated that low detection sensitivity (0.72) was a major limiting factor of using serum IL-6 for PJI diagnosis. Notably, two prior studies that included 103 patients reported poor sensitivity (0.13–0.14) for serum IL-6 to detect periprosthetic shoulder infections^26, 28^. The poor sensitivity reported in these studies may be related to the high proportion of low-grade infections in shoulder PJI. As has been established previously, different microorganisms cause PJI of different joints^6^. Low-virulent bacteria such as *Propionibacterium acnes* are frequently detected in patients with periprosthetic shoulder infection^55^. Nevertheless, Ettinger *et al*. reported a promising result for serum IL-6 testing to predict low-grade PJI with a sensitivity of 0.80^29^. Additional large-scale studies are necessary to determine the diagnostic value of serum IL-6 for shoulder PJI.

Joint aspiration is conventionally performed in patients with suspected PJI. Synovial fluid culture is an accurate diagnosis method with a sensitivity and specificity of 0.72 and 0.95, respectively^33^. Thus, results of aspiration culture can be used to guide treatment plans. In recent years, synovial fluid biomarkers have received significant attention for their diagnostic value for PJI detection. The pooled sensitivities of synovial fluid WCC, polymorphonuclear leukocytes, CRP, α-defensin, and leukocyte esterase are 0.88, 0.90, 0.92, 1.00, and 0.81, respectively, and their pooled specificities are 0.93, 0.88, 0.90, 0.96, and 0.97, respectively^32, 34–36^. Synovial fluid α-defensin appears to be the most valuable test to diagnose PJI (Fig. 5); however, it is not a universal test and is expensive (760 USD per test)^36^. The use of the α-defensin test for PJI diagnosis was first reported in 2014^56, 57^. To our knowledge, it is not yet used in clinical practice in China. The synovial fluid IL-6 test is considerably more common and economical than the synovial fluid α-defensin test. The present meta-analysis indicated that abnormally high synovial fluid IL-6 level is a strong indicator of PJI, with an AUC of 0.96. Notably, our subgroup analysis indicated the high diagnostic value of the synovial fluid IL-6 test was not influenced by the presence of inflammatory diseases, which renders the test more advantageous for PJI detection in clinical practice. However, as with the serum IL-6 test, the optimal cut-off value of synovial fluid IL-6 is yet to be determined (range: 359.3–13,350 pg/mL).

The current meta-analysis has several strengths: 1) to the best of our knowledge, this is the first meta-analysis to report the diagnostic capacity of synovial fluid IL-6 for PJI detection; and 2) when compared to a prior meta-analysis, the current analysis included more studies (11 articles) assessing serum IL-6 test for PJI diagnosis, and showed that serum IL-6 was less sensitive than synovial fluid IL-6. We believe that this meta-analysis may aid orthopaedists to improve the diagnostic accuracy for PJI in clinical practice.

However, this meta-analysis has several limitations. First, the result of statistical analysis indicated significant heterogeneity for the serum IL-6 test. Two studies reported exceedingly low diagnostic values for the serum IL-6 test in the detection of periprosthetic shoulder infections^26, 28^. The heterogeneity was still present after the two studies on shoulder PJI were excluded (I^2^ = 79), which lowered the reliability of the findings. Second, a gold standard test for PJI diagnosis could not be proposed because multiple reference standards were used in the studies included in this meta-analysis. Third, antibiotic therapies and systemic inflammatory diseases may affect the diagnostic accuracy of serum IL-6. Only few studies excluded patients with prior antibiotic therapy or other infections. Fourth, the current study only included articles published between January 1990 and October 2016. Lastly, we did not register a protocol for the current study in PROSPERO.

The results of this meta-analysis showed that synovial fluid IL-6 has significant diagnostic value for PJI diagnosis, particularly in periprosthetic knee and hip infections. Although serum IL-6 test is less sensitive than synovial fluid IL-6 test, it can be regularly prescribed for patients with prosthetic failure owing to its high specificity.

## Methods

The Preferred Reporting Items for Systematic Reviews and Meta-Analyses (PRISMA) statement was used to guide methods for the current study^58^.

### Search Strategy

The MEDLINE and EMBASE databases were searched for studies that utilised the serum or synovial fluid IL-6 test to diagnose PJI between January 1990 and October 2016. The search was based on the following terms: interleukin, IL-6, inflammatory, arthroplasty or replacement, serum or synovial fluid, sensitivity or specificity, septic or septically, prosthesis infection, infectious or infected, and diagnose or diagnostic. Additional entries were identified through the bibliographies of the selected studies and reviews.

The following inclusion criteria were used to evaluate the articles: (1) articles indicated the date of serum or synovial fluid IL-6 assessment of patients undergoing revision arthroplasty; (2) the diagnosis of PJI was made based on the IDSA guideline, Musculoskeletal Infection Society (MSIS) criteria, or American Academy of Orthopaedic Surgeons (AAOS) guideline^8, 45, 46^; (3) sufficient data were described for the calculation of the true-positive (tp), false-negative (fn), false-positive (fp), and true-negative (tn) values; (4) the number of participants were more than 10; and (5) the article was written in English.

When the same patients were included in multiple articles, findings from the most detailed study were used. When an article described more than one cut-off value, the most efficient value was used in the present analysis. After a full-text assessment, article quality was assessed using the QUADAS tool^59^. Two independent reviewers performed the evaluation, and a third reviewer was solicited to resolve disagreements.

### Data Extraction

Two reviewers independently completed data extraction from eligible studies. The characteristics of each study were recorded in a standardised form to include the following details: name of the first author, publication year, number of patients, mean age of patients, study design, type of patient enrolment, location of the operation, cut-off value, and reference standards. A third reviewer was solicited to check for discrepancies and resolve conflicting findings of the first two reviewers.

### Statistical Analysis

Statistical analysis was performed by two independent reviewers. The tp, fn, fp, and tn values were calculated according to the statistical data extracted from eligible articles. Quantitative indicators, including sensitivity, specificity, AUC, and DOR, were calculated to estimate the diagnostic value of the IL-6 test for PJI detection. The PLR, NLR, and post-test probability were used to calculate the clinical utility of the IL-6 test. I^2^ was calculated to evaluate study heterogeneity^60^. Studies with I^2^ values of >50% were considered heterogeneous. For the heterogeneous studies, the diagnostic accuracy of the IL-6 test was calculated using the random effects model^61^. Subgroup analysis was performed to determine the effect of various characteristics on the diagnostic accuracy of the IL-6 test for PJI detection. Publication bias was evaluated using Deeks’ funnel plot asymmetry test^62^. STATA version 14 (StataCorp, College Station, TX, USA) was used for other statistical analyses.

### Data Availability

All data generated or analysed during this study are included in this published article.

## References

[1] Bozic KJ (2009). The epidemiology of revision total hip arthroplasty in the United States. J Bone Joint Surg Am.

[2] Clohisy JC, Calvert G, Tull F, McDonald D, Maloney WJ (2004). Reasons for Revision Hip Surgery. Clinical Orthopaedics and Related Research.

[3] Bozic KJ (2010). The epidemiology of revision total knee arthroplasty in the United States. Clin Orthop Relat Res.

[4] Namba RS, Inacio MC, Paxton EW (2013). Risk factors associated with deep surgical site infections after primary total knee arthroplasty: an analysis of 56,216 knees. J Bone Joint Surg Am.

[5] NIH consensus conference: Total hip replacement. NIH Consensus Development Panel on Total Hip Replacement. *JAMA* **273**, 1950–1956, doi:10.1001/jama.1995.03520480070043 (1995). 7783307

[6] Zimmerli W, Trampuz A, Ochsner PE (2004). Prosthetic-joint infections. N Engl J Med.

[7] Fernandez-Sampedro M (2015). 26Postoperative diagnosis and outcome in patients with revision arthroplasty for aseptic loosening. BMC Infect Dis.

[8] Osmon DR (2013). Diagnosis and management of prosthetic joint infection: clinical practice guidelines by the Infectious Diseases Society of America. Clin Infect Dis.

[9] Berbari E (2010). Inflammatory blood laboratory levels as markers of prosthetic joint infection: a systematic review and meta-analysis. J Bone Joint Surg Am.

[10] Yuan K, Chen HL, Cui ZM (2014). Diagnostic accuracy of C-reactive protein for periprosthetic joint infection: a meta-analysis. Surg Infect (Larchmt).

[11] Song M, Kellum JA (2005). Interleukin-6. Crit Care Med.

[12] van der Poll T (1997). Interleukin-6 gene-deficient mice show impaired defense against pneumococcal pneumonia. J Infect Dis.

[13] Wirtz DC, Heller KD, Miltner O, Zilkens KW, Wolff JM (2000). Interleukin-6: a potential inflammatory marker after total joint replacement. Int Orthop.

[14] Selberg O (2000). Discrimination of sepsis and systemic inflammatory response syndrome by determination of circulating plasma concentrations of procalcitonin, protein complement 3a, and interleukin-6. Crit Care Med.

[15] Di Cesare PE, Chang E, Preston CF, Liu CJ (2005). Serum interleukin-6 as a marker of periprosthetic infection following total hip and knee arthroplasty. J Bone Joint Surg Am.

[16] Bottner F (2007). Interleukin-6, procalcitonin and TNF-alpha: markers of peri-prosthetic infection following total joint replacement. J Bone Joint Surg Br.

[17] Nilsdotter-Augustinsson A (2007). Inflammatory response in 85 patients with loosened hip prostheses: a prospective study comparing inflammatory markers in patients with aseptic and septic prosthetic loosening. Acta Orthop.

[18] Buttaro MA, Tanoira I, Comba F, Piccaluga F (2010). Combining C-reactive protein and interleukin-6 may be useful to detect periprosthetic hip infection. Clin Orthop Relat Res.

[19] Deirmengian C (2010). Synovial fluid biomarkers for periprosthetic infection. Clin Orthop Relat Res.

[20] Worthington T (2010). Serum procalcitonin, interleukin-6, soluble intercellular adhesin molecule-1 and IgG to short-chain exocellular lipoteichoic acid as predictors of infection in total joint prosthesis revision. Br J Biomed Sci.

[21] Jacovides CL, Parvizi J, Adeli B, Jung KA (2011). Molecular markers for diagnosis of periprosthetic joint infection. J Arthroplasty.

[22] Abou El-Khier NT, El Ganainy Ael R, Elgeidy A, Rakha SA (2013). Assessment of interleukin-6 and other inflammatory markers in the diagnosis of Egyptian patients with periprosthetic joint infection. Egypt J Immunol.

[23] Glehr M (2013). Novel biomarkers to detect infection in revision hip and knee arthroplasties. Clin Orthop Relat Res.

[24] Gollwitzer H (2013). Antimicrobial peptides and proinflammatory cytokines in periprosthetic joint infection. J Bone Joint Surg Am.

[25] Deirmengian C (2014). Diagnosing periprosthetic joint infection: has the era of the biomarker arrived?. Clin Orthop Relat Res.

[26] Grosso MJ (2014). Poor utility of serum interleukin-6 levels to predict indolent periprosthetic shoulder infections. J Shoulder Elbow Surg.

[27] Randau TM (2014). Interleukin-6 in serum and in synovial fluid enhances the differentiation between periprosthetic joint infection and aseptic loosening. PLoS One.

[28] Villacis D (2014). Serum interleukin-6 as a marker of periprosthetic shoulder infection. J Bone Joint Surg Am.

[29] Ettinger M (2015). Circulating biomarkers for discrimination between aseptic joint failure, low-grade infection, and high-grade septic failure. Clin Infect Dis.

[30] Frangiamore SJ (2015). Synovial fluid interleukin-6 as a predictor of periprosthetic shoulder infection. J Bone Joint Surg Am.

[31] Frangiamore SJ (2016). Synovial Cytokines and the MSIS Criteria Are Not Useful for Determining Infection Resolution After Periprosthetic Joint Infection Explantation. Clin Orthop Relat Res.

[32] Xie K, Qu X, Yan M (2016). Procalcitonin and alpha-Defensin for Diagnosis of Periprosthetic Joint Infections. J Arthroplasty.

[33] Qu X (2013). Preoperative aspiration culture for preoperative diagnosis of infection in total hip or knee arthroplasty. J Clin Microbiol.

[34] Qu X (2014). Evaluation of white cell count and differential in synovial fluid for diagnosing infections after total hip or knee arthroplasty. PLoS One.

[35] Wang C, Wang Q, Li R, Duan JY, Wang CB (2016). Synovial Fluid C-reactive Protein as a Diagnostic Marker for Periprosthetic Joint Infection: A Systematic Review and Meta-analysis. Chin Med J (Engl).

[36] Wyatt MC (2016). The Alpha-Defensin Immunoassay and Leukocyte Esterase Colorimetric Strip Test for the Diagnosis of Periprosthetic Infection: A Systematic Review and Meta-Analysis. J Bone Joint Surg Am.

[37] Ouyang Z, Li H, Liu X, Zhai Z, Li X (2014). Prosthesis infection: diagnosis after total joint arthroplasty with three-phase bone scintigraphy. Ann Nucl Med.

[38] Xing D (2013). Use of anti-granulocyte scintigraphy with 99mTc-labeled monoclonal antibodies for the diagnosis of periprosthetic infection in patients after total joint arthroplasty: a diagnostic meta-analysis. PLoS One.

[39] Verberne SJ, Raijmakers PG, Temmerman OP (2016). The Accuracy of Imaging Techniques in the Assessment of Periprosthetic Hip Infection: A Systematic Review and Meta-Analysis. J Bone Joint Surg Am.

[40] Tsaras G (2012). Utility of intraoperative frozen section histopathology in the diagnosis of periprosthetic joint infection: a systematic review and meta-analysis. J Bone Joint Surg Am.

[41] Qu X (2013). PCR-based diagnosis of prosthetic joint infection. J Clin Microbiol.

[42] Zhai Z (2014). Meta-analysis of sonication fluid samples from prosthetic components for diagnosis of infection after total joint arthroplasty. J Clin Microbiol.

[43] Ouyang Z (2015). Limitations of Gram staining for the diagnosis of infections following total hip or knee arthroplasty. Exp Ther Med.

[44] Parvizi J, Fassihi SC, Enayatollahi MA (2016). Diagnosis of Periprosthetic Joint Infection Following Hip and Knee Arthroplasty. Orthop Clin North Am.

[45] Della Valle C (2011). American Academy of Orthopaedic Surgeons clinical practice guideline on: the diagnosis of periprosthetic joint infections of the hip and knee. J Bone Joint Surg Am.

[46] Parvizi J (2011). New definition for periprosthetic joint infection: from the Workgroup of the Musculoskeletal Infection Society. Clin Orthop Relat Res.

[47] Tigges S, Stiles RG, Roberson JR (1994). Appearance of septic hip prostheses on plain radiographs. AJR Am J Roentgenol.

[48] Hausfater P (2014). Biomarkers and infection in the emergency unit. Med Mal Infect.

[49] Ferguson-Smith AC (1988). Regional localization of the interferon-beta 2/B-cell stimulatory factor 2/hepatocyte stimulating factor gene to human chromosome 7p15–p21. Genomics.

[50] Helfgott DC (1989). Multiple forms of IFN-beta 2/IL-6 in serum and body fluids during acute bacterial infection. J Immunol.

[51] Sakamoto K (1994). Elevation of circulating interleukin 6 after surgery: factors influencing the serum level. Cytokine.

[52] Barton BE (1997). IL-6: insights into novel biological activities. Clin Immunol Immunopathol.

[53] Gauldie J, Richards C, Harnish D, Lansdorp P, Baumann H (1987). Interferon beta 2/B-cell stimulatory factor type 2 shares identity with monocyte-derived hepatocyte-stimulating factor and regulates the major acute phase protein response in liver cells. Proc Natl Acad Sci USA.

[54] Povoa P (2002). C-reactive protein: a valuable marker of sepsis. Intensive Care Med.

[55] Sperling, J. W., Kozak, T. K., Hanssen, A. D. & Cofield, R. H. Infection after shoulder arthroplasty. *Clin Orthop Relat Res* 206–216, doi:10.1097/00003086-200101000-00028 (2001). 10.1097/00003086-200101000-00028 11153989

[56] Deirmengian C (2014). Combined measurement of synovial fluid alpha-Defensin and C-reactive protein levels: highly accurate for diagnosing periprosthetic joint infection. J Bone Joint Surg Am.

[57] Bingham J (2014). The alpha defensin-1 biomarker assay can be used to evaluate the potentially infected total joint arthroplasty. Clin Orthop Relat Res.

[58] Liberati A (2009). The PRISMA statement for reporting systematic reviews and meta-analyses of studies that evaluate health care interventions: explanation and elaboration. J Clin Epidemiol.

[59] Whiting P, Rutjes AW, Reitsma JB, Bossuyt PM, Kleijnen J (2003). The development of QUADAS: a tool for the quality assessment of studies of diagnostic accuracy included in systematic reviews. BMC Med Res Methodol.

[60] Higgins JP, Thompson SG, Deeks JJ, Altman DG (2003). Measuring inconsistency in meta-analyses. BMJ.

[61] Gardiner JC, Luo Z, Roman LA (2009). Fixed effects, random effects and GEE: what are the differences?. Stat Med.

[62] Deeks JJ, Macaskill P, Irwig L (2005). The performance of tests of publication bias and other sample size effects in systematic reviews of diagnostic test accuracy was assessed. J Clin Epidemiol.

